# Phylogenetic analysis of the complete mitochondrial genome of *Sargassum patens C. Agardh* (Phaeophyceae)

**DOI:** 10.1080/23802359.2020.1841580

**Published:** 2020-12-24

**Authors:** Jingjing Li, Yuanxin Bi

**Affiliations:** a Key Laboratory of Marine Hazards Forecasting, Ministry of Natural Resources, Hohai University, Nanjing, China; b Key Laboratory of Sustainable Utilization of Technology Research for Fishery Resource of Zhejiang Province, Marine Fisheries Research Institute of Zhejiang, Zhoushan, China

**Keywords:** *Sargassum patens*, Mitochondrial genome, sargassaceae

## Abstract

The mitochondrial genome of *Sargassum patens* was completely sequenced. This mitogenome is a circular molecule of 34,844 bp in length and had an overall GC content of 36.20%. The mitogenome contains 37 protein-coding, three rRNA, 25 tRNA genes and two conserved open reading frames (ORFs). The Maximum-likelihood (ML) tree showed that *S. patens* has a closer relationship with *S. aquifolium*.

*Sargassum patens* C. Agardh (Sargassaceae, Phaeophyceae) is a perennial macroalgae, known widely distributed on rocky subtidal shores from the Japanese and Chinese seas (Huang et al. 2013). The detached thalli of *S. patens* had a floating period of between 1 and 9 weeks (Yatsuya 2008), which could provide food and habitat for fish larva and invertebrates in pelagic ecosystem (Yamasaki et al. 2014). We described the characterization of complete mitogenome of *S. patens* which were expected to facilitate future studies on taxonomic resolution, population genetic structure and phylogenetic relationships.

The specimen was collected from Bailong Island, Guangxi Province, China (21°30′20″N; 108°13′46″E) on 20 January 2020, and stored at the Marine Biological Museum, Chinese Academy of Sciences with an accession number MBM286786. Paired-end libraries with insert sizes of ∼400 bp were prepared and used for Illumina HiSeq 2000 sequencing. The raw paired end reads were trimmed and quality controlled by Trimmomatic (Bolger et al. 2014). ABySS was used to perform genome assembly (Simpson et al. 2009), and GapCloser software (https://anaconda.org/bioconda/soapdenovo2-gapcloser) was applied to fill up the remaining local inner gaps and corrected the single base polymorphism. The transfer RNA (tRNA) genes were predicted by tRNAscan-SE (Lowe and Chan 2016) and the ribosomal RNA (rRNA) genes and protein coding genes were determined based on homology alignments with *Sargassum aquifolium* (NC033408). To explore the phylogenetic position of *S. patens*, mitochondrial genome sequences of 16 species (downloaded from the GenBank database) were used to construct phylogenetic tree by the Maximum-likelihood (ML) method, with *Turbinaria ornata* (GenBank accession number KM501562) served as the out-group.

The complete mitogenome (MT740313) comprises a circular DNA molecule measuring 34,844 bp in length with 36.20% overall GC content. The mitogenome contains 65 genes, including 37 protein-coding, three rRNA, 25 tRNA genes and two conserved open reading frames (ORFs). All protein-coding genes use the start codon ATG. Of the 37 protein-coding genes, 27 ended with the TAA stop codon, five with TAG and five with TGA. The lengths of three rRNA genes are 1939 bp (rnl rRNA), 874 bp (rns rRNA), and 126 bp (rrn5 rRNA). All genes show the typical gene arrangement conforming to the Sargassaceae family consensus (Liu et al. 2019). Phylogeographic tree showed that *S. patens* clustered together with *S. aquifolium* (Figure 1). The complete mitogenome sequence provided herein would help understand molecular evolution of the related species of Sargassaceae.

**Figure 1.**
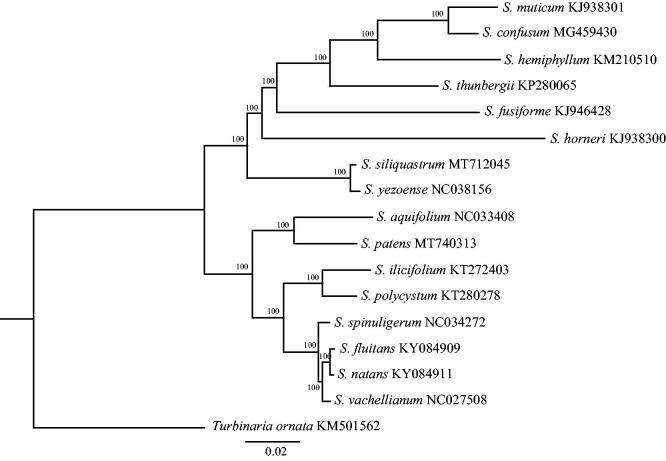
Maximum-likelihood phylogenetic tree based on *S. patens* and 16 other mitochondrial genomes of Sargassaceae family in the NCBI.

## Data Availability

The data that support the findings of this study are openly available in repository under Accession: MT740313. https://www.ncbi.nlm.nih.gov/nuccore/1898549977. Raw sequence data were submitted to the NCBI SRA (BioProject: PRJNA668679).
